# Exploring factors for melodic diversification of folk songs in the Ryukyu Archipelago

**DOI:** 10.1017/ehs.2025.10010

**Published:** 2025-07-22

**Authors:** Yuri Nishikawa, Yasuo Ihara

**Affiliations:** 1 Department of Biological Sciences, The University of Tokyo, Bunkyo-ku, Tokyo, Japan; 2 Department of Molecular Life Science, Tokai University School of Medicine, Isehara-shi, Kanagawa, Japan

**Keywords:** cultural evolution, evolution of music, folk songs, melodic variation, Ryukyu Archipelago

## Abstract

Cultural evolution of traditional music around the world has been the subject of recent quantitative investigations. Researchers have explored cultural diffusion of music as well as patterns of geographic variation that may result. By comparison, less has been studied about the process of music diversification; in particular, under what circumstances music diversifies is yet to be understood. In this study, we examine possible factors that may facilitate music diversification, using data from folk songs in the Ryukyu Archipelago, south-western islands of Japan. For a quantitative analysis, we first transform the melody of each folk song, following an automated scheme, into a sequence of alphabets, which is then used to quantify the melodic dissimilarity between each pair of songs. Our particular interest is in the dissimilarity between putative sister songs, or songs that are inferred to have derived from a common origin, and factors that have positive or negative effects on it. Our results suggest that sister songs tend to diversify more when they are sung in different islands, probably as a result of one being transmitted from one island to another, and when they have come to be sung in different social contexts.

## Graphical abstract

**Figure:**
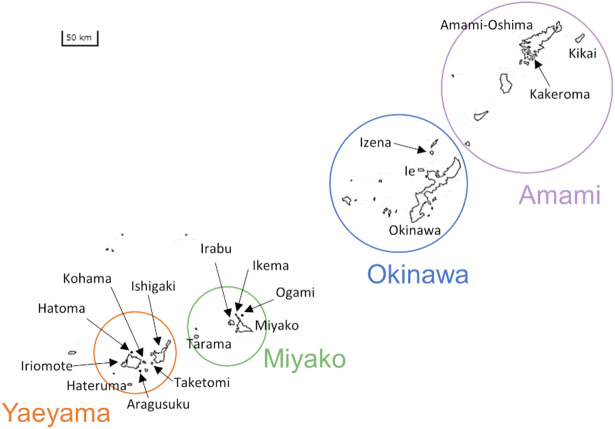

## Social media summary

Folk songs in the Ryukyu Archipelago, Japan, diversify more when sung in different islands or social contexts

## 1. Introduction

A growing number of studies have investigated the diversity of traditional music around the world using quantitative methods (Mehr et al., 2019; Passmore et al., 2024; Savage, 2019; Wood et al., 2022). Although these studies have illuminated patterns of music diversity, less attention has been paid to under what circumstances the diversification is likely to occur. For example, Nishikawa and Ihara (2022) analysed the geographic variation of folk songs in the Ryukyu Archipelago of Japan using a cultural evolutionary approach, and suggested that horizontal transmission between regions played a major role in shaping the observed patterns of folk song diversity; however, the sort of factors that facilitated the diversification process remain unexplored.

In this study, we aim to examine possible factors that may have promoted diversification of folk songs. For that purpose, we focus on groups of folk songs in the Ryukyu Archipelago that are inferred to have derived from a single origin and evaluate the effects of possible factors on the diversity within those groups. In each region of the Ryukyu Archipelago, folk songs developed under the influence of Ryukyuan classical music of the noble class of the Ryukyu Kingdom from the fifteenth century onwards and respective local traditions (Yamanouchi, 1959; Uchida, 1983; Nippon Hoso Kyokai [NHK], 1989–1993). NHK, the Japanese public broadcasting company, has collected recordings of these folk songs from around the archipelago. Traditionally, songs have been transmitted orally, but recently they may also be learnt by listening to recordings. When there is accompaniment to songs, the most commonly used instrument throughout the archipelago is the sanshin, a stringed instrument. Accompaniment by the sanshin is sometimes transcribed by adapting musical notation used in Ryukyuan classical music, called kunkunshi (NHK, 1989–1993).

Within the Ryukyu Archipelago, there are cases in which a pair of folk songs exhibit a close similarity despite being sung in distant islands, and other cases in which songs are quite dissimilar to each other despite having the same title (NHK, 1989–1993). Ethnomusicologists have attempted to disentangle the complex history of the folk songs in the Ryukyu Archipelago, and proposed a shared origin for some groups of songs based on a general judgement of similarities in melodies, lyrics, and titles (NHK, 1989–1993). One objective of the present study is to examine whether these qualitative identifications of sister songs are supported by a simplified quantitative method based strictly on melodic similarity. Another is to infer the roles of geography, time, and social context in the process of song diversification by evaluating the effects of these factors on the diversity within closely related songs.

Regarding the effect of geography, cultural transmission of songs within a narrow geographic area may occur through repeated opportunities of singing and hearing, and if so, the transmission is expected to be relatively accurate. By comparison, transmission of songs over a long distance may result from a single or a few instances of singing/hearing, when the singers or hearers travel somewhere distant from their places of residence, in which case, the transmission is expected to be more error-prone. In particular, the isolation between islands by the sea is expected to have a large effect on song diversification, for the same reason that the islands in the Ryukyu Archipelago exhibit rich biodiversity and endemism (Chiang & Schaal, 2006; Motokawa, 2000; Ota, 1998). As for time, we investigate possible effects of temporal changes in melodies in a given place (or to put it differently, the amount of mutation in the vertical transmission of melodies) by taking into consideration the difference of the recording years of songs. Finally, we focus on the difference in social contexts in which the folk songs are sung. Nishikawa and Ihara (2022) demonstrated the importance of social context in song transmission by showing that songs sung in the ‘work’ context exhibited larger divergence between regions than those sung in other contexts, and that the variation in the work songs are associated with the linguistic variation within the Ryukyu Archipelago. Therefore, we expect that sister songs diversify more when they come to be sung in different social contexts.

Our focus on melody is motivated by the results of a previous study. Nishikawa and Ihara (2022) measured the distances between 1,342 folk songs in the Ryukyu Archipelago using CantoCore (Savage et al., 2012), a cross-cultural music classification scheme. In their multidimensional scaling (MDS) plots, songs did not cluster according to their geographical locations or the social contexts in which they were sung, but did form two clusters roughly corresponding to ‘a-modal’ songs (which do not include pitch classes at a minor or major third above the tonic) and ‘major iso-modal’ songs (which have major third notes but lack minor third notes). This result suggests the significance of musical scales in the diversity of folk songs in the Ryukyu Archipelago. Therefore, in this study, we concentrate on the variation in melodies as well as the scales on which they are based.

**Figure 1.**
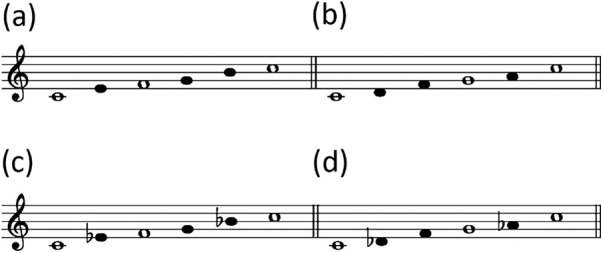
Four types of scales that constitute traditional Japanese songs advocated by Koizumi (1958). (a) The Ryukyu scale. (b) The ritsu scale. (c) The minyo scale. (d) The miyako-bushi scale. Notes indicated in white are considered to be important as ‘nuclear tones’. Based on the figures by Koizumi (1958) and NHK (1989–1993).

Koizumi (1958) advocated four types of basic scales that constitute traditional Japanese songs (Figure 1), and most of the subsequent studies are based on this theory. The Ryukyu scale is found in the Ryukyu Archipelago (especially from Okinoerabu island in the Amami islands in the north to the Yaeyama islands in the south) and Indonesia. The ritsu scale is broadly found in East Asia including the Ryukyu Archipelago. The minyo scale is very common in mainland Japan but infrequent in the Ryukyu Archipelago, except in the northern part of the Amami islands, where it is relatively popular. The miyako-bushi scale is also found in mainland Japan and rare in the Ryukyu Archipelago, but it is found in the northern part of the Amami islands (Koizumi, 1958; NHK, 1989–1993).

Melodies are suitable for microevolutionary approaches, as they can be represented as sequences of notes analogous to DNA or protein sequences (Savage, 2019). Recently, based on such ideas, studies have been conducted to quantify the similarity between melodies of various kinds of music (Bountouridis et al., 2017; Hillewaere et al., 2014; Janssen et al., 2017; Mongeau & Sankoff, 1990; Mora et al., 2016; Savage et al., 2022; van Kranenburg et al., 2013). Savage and Atkinson (2015) developed a method for quantifying the similarity between melodies by coding and aligning them as sequences of the 12 pitch classes. They proposed combinations of parameter values for their sequence alignment algorithms that perform best when separating songs into different tune families identified by expert musicologists, and when measuring similarities between songs within tune families. Note that a tune family is defined by Bayard (1950) as ‘a group of melodies showing basic interrelation by means of constant melodic correspondence, and presumably owing their mutual likeness to descent from a single air that has assumed multiple forms through processes of variation, imitation, and assimilation’. In what follows, we quantify the difference between melodies of folk songs in the Ryukyu Archipelago using Savage and Atkinson’s (2015) method. Then we examine whether the qualitative classification of sister songs based on a general judgement is supported by the quantitative evaluation of melodic similarity. Finally, we evaluate the effects of various factors on the variation of melodies within presumably related songs to infer under what circumstances music diversification is accelerated.

## 2. Materials and methods

### 2.1. Data

**Figure 2.**
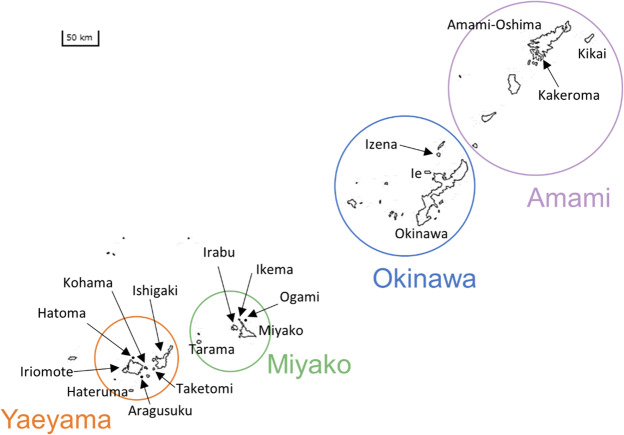
Map of the Ryukyu Archipelago. The locations of the four regions and 17 islands used for the analyses are indicated. Created based on a map from Geospatial Information Authority of Japan (https://maps.Gsi.Go.Jp/vector/).

We used two sources of data on folk songs in the Ryukyu Archipelago of Japan. First, melodies were sampled from published musical scores in ‘A Survey of Japanese Folksongs – Okinawa-Amami Islands’ (hereafter SJF; NHK, 1989–1993), for which songs were recorded between 1964 and 1990. Songs in SJF were collected by NHK, with an intention to select songs reflecting traditional life and culture of each region and to include various types of songs (NHK, 1989–1993). Second, for the purpose of examining the possible effect of temporal changes in melodies, we used audio recordings of songs sung by Ryukyuan singers, which were collected by one of the authors (Y. Nishikawa) between 2015 and 2019. In SJF, some songs are similar overall to each other and thus are assumed to have the same origin. As for the songs from our original recordings, the singers described some of the songs they sang as being closely related to others. On the basis of these descriptions, one of the authors (Y. Nishikawa) compiled the songs from SJF and our original recordings into ‘song groups’, or groups of songs that are inferred to have derived from a common origin. Of all audio recordings that we collected, those melodies that did not share the same song group with any melodies from SJF were excluded. For our main analysis, we used 38 song groups (Supplementary Table S1) that included at least three songs, whether from SJF or our original recordings, which amounted to 148 songs from four regions in the Ryukyu Archipelago: Amami, Okinawa, Miyako, and Yaeyama (Figure 2). Eighty-eight of these songs were from SJF, and the remaining 60 were from the audio recordings. In addition, we repeated the analysis using only 44 songs from SJF that belonged to 13 song groups including at least three SJF songs. The songs analysed were old songs whose lyricists and composers are unknown. Songs written in the twentieth century by known lyricists and composers, called shin minyo (new folk songs), were excluded. Ryukyuan classical music, in which variants are not allowed, were also excluded. On the other hand, melodies from SJF (O2.2, O11.1, O12.1, O13.1) that had been published in commercially available records were analysed without distinction from other melodies. Melodies from the audio recordings were performed by professional or amateur singers (it is often difficult to clearly distinguish between them) and were analysed without distinction. Our choice of not excluding songs with possible commercial influence is generally in line with Pendlebury’s (2020) view that the distinction between commercial and folk music is not straightforward.

### 2.2. Coding

**Figure 3.**
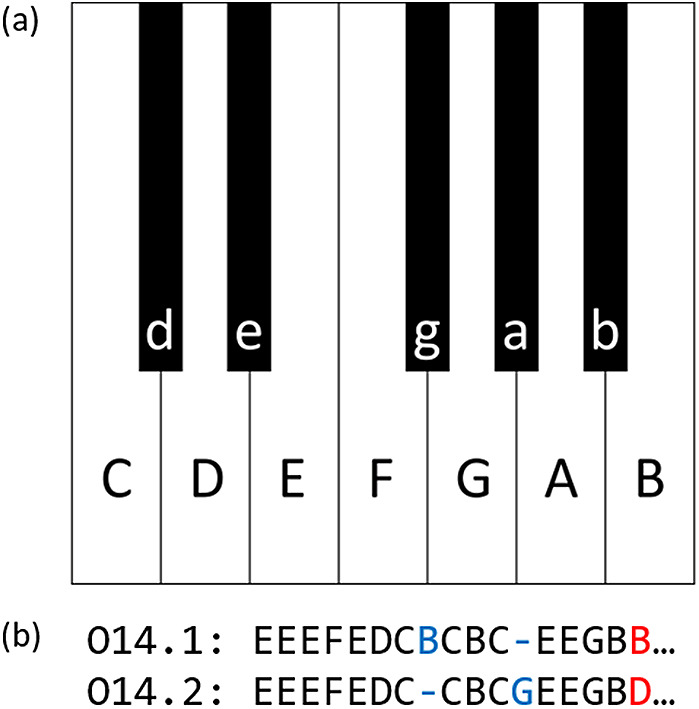
(a) Letters assigned to the 12 pitch classes. (b) Example of alignment of a pair of melodies using parameter set 2.

Following Savage and Atkinson (2015), the melody of each song was coded as a sequence of alphabets assigned to the 12 pitch classes as shown in Figure 3a, where note values and octave differences were ignored. Although alternative coding methods can also be applied, previous studies have produced mixed results about whether the inclusion of note durations improves alignment performance (Janssen et al., 2017; van Kranenburg et al., 2009). Therefore, this study used a conventional coding method that can deal with melodies as simply as possible, that is, ignoring note durations (Bountouridis et al., 2017; Savage et al., 2022) and octaves (Bountouridis et al., 2017; Mongeau & Sankoff, 1990; Savage et al., 2022). All melodies were transposed so that the tonic was C, and repeated parts and responsorial parts were omitted. The audio recordings were coded with the help of WaveTone (https://ackiesound.ifdef.jp/), a free software program for audio analysis. Folk songs in Japan including the Ryukyu Archipelago are not created based on 12-tone equal temperament, but are based on the four types of five-tone scales (Figure 1) and conventionally represented approximately in staff notation (Koizumi, 1958). As this study does not analyse pitch difference, there are no major problems in assigning alphabets of the 12 pitch classes.

### 2.3. Alignment and similarity between melodies

To infer shared origin between melodies, we aligned the coded sequences following Savage and Atkinson (2015). In particular, we used two sets of parameter values proposed by Savage and Atkinson (2015) for the gap opening penalty (GOP), the gap extension penalty (GEP), and whether difference in mode is included or ignored (i.e. whether lowercase letters are recoded as uppercase letters). The GOP and GEP are to penalize the creation and extension of a gap in a sequence, where the smaller the penalties are, the more likely gaps are to be inserted in the alignment. Small penalties are adequate for closely related sequences, and large penalties are adequate for more divergent sequences (Thompson et al., 1994). Parameter set 1 (GOP = 12, GEP = 6, ignoring mode) is suggested to be suitable for separating between ‘tune families’, that is, groups of songs that are known to be closely related to each other. Parameter set 2 (GOP = 0.8, GEP = 0.2, including mode) is considered suitable for sequence alignment within a tune family (Figure 3). The similarity between two melodies was calculated as percent identity (PID) according to the following formula:

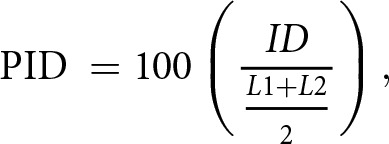


where ID represents the number of identical notes after alignment, and L1 and L2 are the lengths of sequences prior to alignment. These analyses were conducted in R version 4.1.1 applying code available at https://github.com/pesavage/melodic-evolution.

### 2.4. Neighbor-Net

To examine whether the song groups identified by a general judgement can be reproduced by a phylogenetic analysis based on the automated quantification of melodic similarities, we performed a Neighbor-Net analysis with the distance between melodies being calculated as 1 − PID/100. A network was obtained for each of the four regions (Figure 2) and each parameter set, using SplitsTree4 (Huson & Bryant, 2006).

### 2.5. Linear mixed model

A linear mixed model (LMM) analysis was performed to examine the factors affecting the melodic changes using lme4 and lmerTest packages in R version 4.1.1. The dependent variable was the distance between 320 pairs of melodies belonging to the same song group, calculated as 1 − PID/100. We first constructed the following full model that included all the independent variables that we considered to be possible factors producing the distance between melodies:
(1)
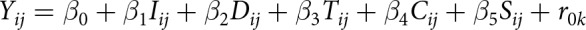


where *Y_ij_* is the difference between melodies *i* and *j* belonging to song group *k*.

The independent variables are as follows. First, *I_ij_* indicates whether melodies *i* and *j* were recorded in different islands (0 if the same island, 1 if different islands). Second, *D_ij_* denotes the geographic distance between the sites where melodies *i* and *j* were recorded. For the geographic distance, the latitudes and longitudes of the recording sites were obtained from the documented names of the places using the CSV Address Matching Service (https://geocode.csis.u-tokyo.ac.jp/) provided by the Center for Spatial Information Science at the University of Tokyo, except for two cases (song ID A9.1 and M3.4 in Supplementary Table S1), in which the longitudes and latitudes were obtained using the map from the Geographical Survey Institute of Japan (https://maps.gsi.go.jp/). The geographic distances (km) between locations were calculated from the longitudes and latitudes using geosphere package in R version 4.1.1. As some melodies were described as being sung throughout the Okinawa islands (song ID O2.2, O11.1, O12.1, O13.1; NHK, 1989–1993) and there is no information on where they were recorded, *I_ij_* and *D_ij_* between these and other melodies were set to NA. These variables are included to capture the geographic effects on the amount of mutation in the horizontal transmission of melodies. In other words, as *I_ij_* changes from 0 to 1 or *D_ij_* increases, *Y_ij_* is expected to increase as well.

Third, *T_ij_* represents the difference of the recording years between melodies *i* and *j*. This variable is intended to measure the amount of mutation in the vertical transmission of melodies. In other words, as *T_ij_* increases, *Y_ij_* is expected to increase as well. Fourth, *C_ij_* indicates whether melodies *i* and *j* were sung in different social contexts or not (0 if the same context, 1 if different contexts). SJF classified songs into ‘child’, ‘ritual’, ‘work’, and ‘amusement’ songs according to the social context in which they were sung in village communities of the Ryukyu Archipelago based on a scheme partially derived from Yanagita (1940). The social context ‘child’ includes lullabies and songs sung by children. Melodies of ‘ritual’ analysed in this study are sung for festivals, rain-making, and so on. Melodies of ‘work’ analysed in this study are sung in farming, shipbuilding, and so on. Melodies of ‘amusement’ are sung for the sake of singing. Similarly, we determined the social contexts of our originally recorded songs based on descriptions of the songs by the singers. We examine the hypothesis that sister melodies are more dissimilar when they come to be sung in different social contexts. In other words, as *C_ij_* changes from 0 to 1, *Y_ij_* is expected to increase as well. Fifth, in order to control for any possible difference between melodies from SJF and those from our original recordings, we included *S_ij_*, which indicates whether the sources of melodies *i* and *j* are different (0 if the same source, 1 if different sources). These independent variables are listed in Table 1. Finally, to control for possible difference between song groups in the rate of melodic change, a random effect of song groups on the intercept (*r*_0*k*_) was included. Song groups may vary in, for example, the number of notes with strong rhythmic functions, which has been suggested to be negatively associated with the rate of change (Savage et al., 2022).

**Table 1.** List of the independent variables of LMM

Variable	Description	Expected effect
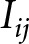	Whether melodies *i* and *j* were recorded in different islands (1) or not (0)	Positive
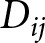	Geographic distance between the sites where melodies *i* and *j* were recorded	Positive
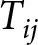	Difference of the recording years between melodies *i* and *j*	Positive
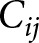	Whether melodies *i* and *j* were sung in different social contexts (1) or not (0)	Positive
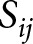	Whether the sources of melodies *i* and *j* are different (1) or not (0)	None

Model selection was performed using the step function of lmerTest package. First, starting from the full model (Eq. (1)), backward elimination of the random effects was performed using a likelihood ratio test with a significance level of 0.1. This was followed by backwards elimination of the fixed effects using an F-test based on Satterthwaite’s approximation with a significance level of 0.05. Backwards elimination was chosen to avoid overlooking important variables by accepting too-simplistic models (Kuznetsova et al., 2017, 2015).

## 3. Results

### 3.1. Similarity between melodies

**Figure 4.**
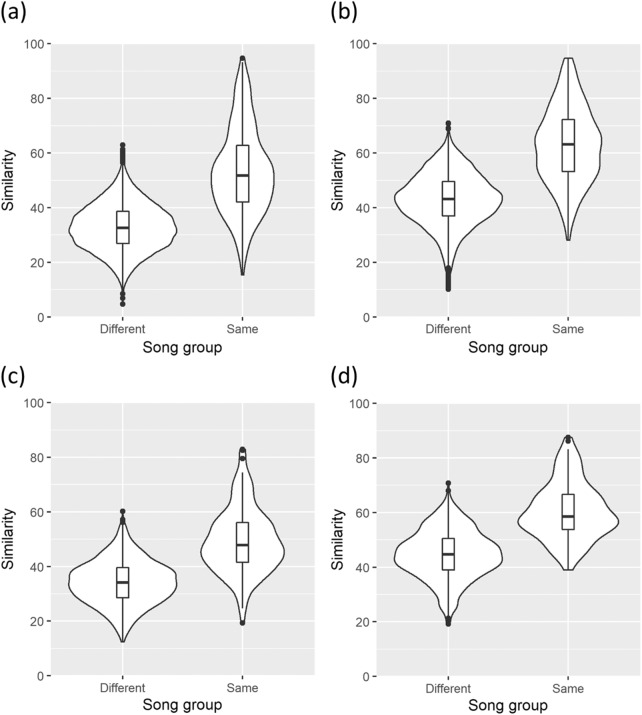
Distributions of PID between melodies from different song groups and the same song groups. PID values were calculated using (a) all melodies and parameter set 1, (b) all melodies and parameter set 2, (c) only melodies from SJK and parameter set 1, and (d) only melodies from SJK and parameter set 2. The boxes represent the first, second and third quartiles, and lengths of the whiskers represent 1.5 × IQR.

PID between melodies of the same song group tended to be high. When parameter set 1 was used, the median PID between melodies from the same song groups was 51.75 (interquartile range [IQR] 42.07–62.77) and the median PID between melodies from different song groups was 32.63 (IQR 26.87–38.66) (Figure 4a). When parameter set 2 was used, the median PID was 63.21 (IQR 53.27–72.29) for the same song groups and 43.18 (IQR 36.92–49.56) for different song groups (Figure 4b). When limited to melodies from SJF and using parameter set 1, the median PID was 47.86 (IQR 41.48–56.14) for the same song groups and 34.09 (IQR 28.57–39.53) for different song groups (Figure 4c). When parameter set 2 was used, the median PID was 58.58 (IQR 53.73–66.67) for the same song groups and 44.71 (IQR 38.96–50.51) for different song groups (Figure 4d). In all four cases, the Brunner–Munzel test, which is a nonparametric rank test for two distributions without assuming equal variances (Brunner & Munzel, 2000), confirmed that the distributions of the PID values are different between the same-group and different-group comparisons (*p* < 0.01). These results suggest that quantitative evaluation of song relationships based solely on melodic similarities largely supports the holistic and potentially more subjective classification of song groups.

### 3.2. Neighbor-Net

**Figure 5.**
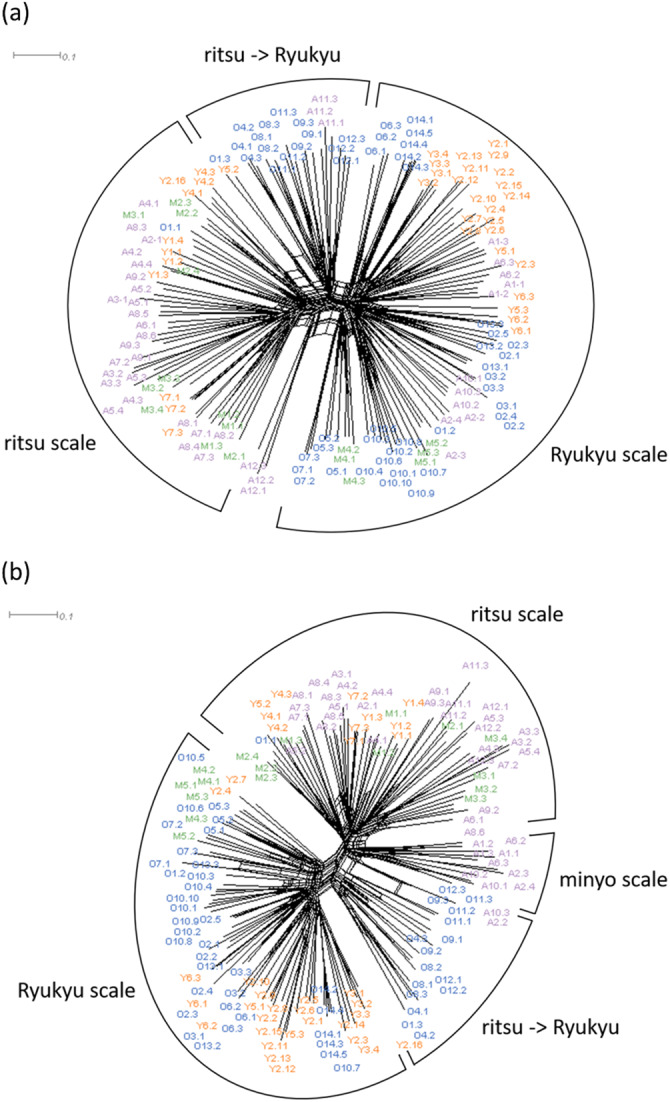
Neighbor-Net graphs based on the distances between melodies of the entire Ryukyu Archipelago with (a) parameter set 1 (*δ* = 0.407) and (b) parameter set 2 (*δ* = 0.3575). Colours indicate the regions corresponding to Figure 2.

Neighbor-Net graphs of melodies are shown in Figure 5 and Supplementary Figures S1–4. Delta score (δ) is a measure of deviation from tree structure of phylogenetic data, which equals zero if the data are perfectly consistent with a tree structure and otherwise ranges between 0 and 1. When the delta score is large, the relationship of the taxa is more appropriately represented by a network rather than a tree (Holland et al., 2002). As this study does not assume a single ancestor and phylogenetic relationship between all the melodies analysed, it is reasonable that δ showed moderately large values. Song IDs shown in these figures specify songs as well as the song groups to which they belong; for example, song ID ‘A1.1’ represents the first song in song group A1 (Supplementary Table S1). In the networks of the entire Ryukyu Archipelago, melodies of the same song groups basically formed clusters, except for some melodies of the song groups A2-8, O1, M2, Y2, and Y5. Of these exceptional song groups, the social contexts of the melodies in A2-6 and O2 were varied within a group. This finding further supports the notion that the quantitative evaluation of melodic similarity in songs is congruent with the qualitative classification of song groups (Figure 5). Clusters were formed by the songs based on the Ryukyu and ritsu scales rather than the songs from different regions for both parameter sets 1 and 2. This may be because melodies belonging to the same song group are likely to retain the same mode and the combinations of alphabets used in melodies are the same for the same scale. Although melodies based on the minyo scale (song ID A1.1-3, A2.2-4, A6.2, A6.3, A10.1-3), all of which were from Amami, were included in the cluster of the Ryukyu scale for parameter set 1 (Figure 5a), this is as expected because parameter set 1 ignores difference in mode, and as a consequence, does not distinguish the Ryukyu and minyo scales. Indeed, when parameter set 2 was used, the melodies based on the minyo scale formed a single cluster adjacent to the cluster of the ritsu scale (Figure 5b). In addition, some melodies based on the Ryukyu scales of Okinawa (song ID O1.3, O4.1-3, O8.1-3, O9.1-3, O11.1-3, O12.1-3) were separated from the Ryukyu cluster for both parameter sets. This may be consistent with the claim that song groups O4, O8, O9, and O12 (see Supplementary Table S1) were originally in the ritsu scale and have transformed to be in the Ryukyu scale (NHK, 1989–1993).

There are some interesting findings from networks of melodies drawn separately for each region. In the networks of Amami, melodies sung in the social context of ritual (song ID A2.1, A2.2, A3.1, A4.1, A4.2, A4.4, A5.1, A5.2, A6.1, A7.1, A7.2, and A7.3) did not show a clear cluster but were roughly cohesive (Supplementary Figure S1). In the networks of Okinawa, songs belonging to a group called ‘Myakuni’ (song group O10) formed a cluster (Supplementary Figure S2a). These are lyrical songs sung in various areas of the Okinawa islands, without fixed lyrics and sometimes improvised (NHK, 1989–1993). Despite the large variety of lyrics, it was confirmed that similar melodies had been maintained across broad areas. It is also said that the melodies of ‘Myakuni’ are similar to those of ‘Tarama-shunkani’ (song group M5) sung in Miyako (NHK, 1989–1993), and indeed they are close to each other in the network of the entire Ryukyu Archipelago with parameter set 1. ‘Tarama-shunkani’, which clustered together in the networks of Miyako (Supplementary Figure S3), are lyrical songs about farewell to an official leaving an island by his local wife. In the networks of Yaeyama, rain-making songs (song group Y2) were divided into two clusters for both parameter sets except for song ID Y2.16, but the clusters split differently depending on the parameter sets (Supplementary Figure S4). Based on previous ethnomusicological studies, rain-making songs in Yaeyama are considered to be further classified into two types (NHK, 1989–1993). One type has diverse melodies and beats, and the tempo is generally slow, whereas the other has less diverse melodies and is in clear duple time, with the tempo being faster. The clusters observed in the Neighbor-Net graphs did not perfectly match this classification, possibly because beat and tempo were not included in the analysis; however, these may suggest that the two types of melodies have not evolved from two clearly distinct ancestral melodies.

### 3.3. Linear mixed model

Residual and QQ plots for the full model (Eq. (1)) are shown in Supplementary Figure S5. Because the assumptions of linearity and normality are largely satisfied, it is appropriate to use the LMM. As the following results were almost the same between using parameter set 1 and 2, only the results using parameter set 2 are shown in the main article, and the results using parameter set 1 are shown in the Supplement. Model selection was performed using standardized independent and dependent variables, as a result of which the following model was obtained for both parameter sets 1 and 2 (Supplementary Table S2ab):
(2)
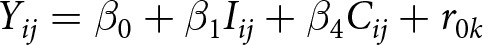


In model (2), the variance of the random effect was slightly larger than the variance of the residuals, indicating a stronger random effect of song groups (Supplementary Table S4). The estimated partial regression coefficients for model (2) are shown in Table 2 and Supplementary Table S5. For both parameter sets, the effects of difference in islands and social contexts were statistically significant, with the standard partial regression coefficient for difference in social contexts being larger than that for difference in islands.

**Table 2.** Results of LMM analysis for all melodies. Model (2) with standardized variables and parameter set 2

	Estimate	Std. error	df	*t* value	Pr(>|*t*|)	
(Intercept)	−0.284	0.133	35.231	−2.136	0.040	*
Island	0.123	0.049	296.019	2.525	0.012	*
Social context	0.277	0.054	302.123	5.133	5.12E−07	***

Signif. codes: 0

‘***’ 0.001 ‘**’ 0.01 ‘*’ 0.05 ‘.’ 0.1 ‘’ 1.

Whereas standardized variables were used to compare the effect sizes across independent variables, non-standardized variables were used to quantify the amount of dissimilarity of melodies corresponding to changes in each independent variable (e.g. how much the melody changes when two recording sites are 1 km apart from each other). Model selection based on non-standardized independent and dependent variables resulted in the same model as model (2) for both parameter sets (Supplementary Table S2ef). The estimates of partial regression coefficients are shown in Table 3 and Supplementary Table S6. The estimates of the effect of difference in islands were 0.044 for parameter set 1 and 0.034 for parameter set 2, representing the extra amount of dissimilarity between a pair of melodies from the same song group when they had been recorded in different islands as compared with when they were from the same island. The estimates of the effects of difference in social contexts were 0.164 for parameter set 1 and 0.145 for parameter set 2, showing the extra distance between a pair of melodies from the same social group when they are sung in different social contexts as compared with when sung in the same social context. Additional analyses using several models not selected in the model selection were also conducted (Supplementary Table S2cdgh). In any cases, the effect of geographic distance and difference in recording years were not significant (Supplementary Tables S8–10).

**Table 3.** Results of LMM analysis for all melodies. Model (2) with non-standardized variables and parameter set 2

	Estimate	Std. error	df	*t* value	Pr(>|*t*|)	
(Intercept)	0.307	0.019	38.258	16.463	5.93E–19	***
Island	0.034	0.014	296.019	2.525	0.012	*
Social context	0.145	0.028	302.123	5.133	5.12E−07	***

Signif. codes: 0

‘***’ 0.001 ‘**’ 0.01 ‘*’ 0.05 ‘.’ 0.1 ‘’ 1.

Although the model selection did not indicate a significant effect of the difference in sources of melodies (*S*), we conducted additional analyses using only melodies from SJF (126 pairs of melodies from the same song group), considering the potential difference in data characteristics, such as coding criteria, between SJF and our original recordings. Model selection based on standardized variables obtained the following model for both parameter sets (Supplementary Table S3ab):
(3)
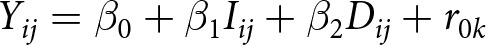


Other independent variables than *I* and *D* were eliminated by the model selection. The estimates of partial regression coefficients are shown in Table 4 and Supplementary Table S7. For both parameter sets, the effect of difference in islands was significantly positive, and the effect of geographic distance was significantly negative. The negative effect of geographic distance is contrary to our hypothesis, which posits that because cultural transmission of melodies within a narrow geographic area may occur repeatedly, melodies derived from the same origin tend to remain similar. The result is difficult to interpret and might be an artefact attributable to the small sample size and a positive correlation between *I* and *D* (*r* = 0.584, *p* < 0.01), even though multicollinearity was not suggested (Supplementary Table S13). When model selection was made under the restriction that at most one of *I* and *D* is entered into the model, a model without any fixed effect was selected for both parameter sets (Supplementary Table S3c–f). In contrast to model (2), the effect of difference in social context was not included in model (3). According to a post-hoc power analysis for the full model using the powerSim function in the simr package, the statistical power for difference in social contexts was high (99.90%) for both parameter sets when all melodies were used, but lower (19.70% for parameter set 1 and 13.40% for parameter set 2) when limited to melodies from SJF, probably due to the small sample size.

**Table 4.** Results of LMM analysis for melodies from SJF. Model (3) with standardized variables and parameter set 2

	Estimate	Std. error	df	*t* value	Pr(>|*t*|)	
(Intercept)	−0.019	0.281	11.392	−0.066	0.948	
Island	0.281	0.096	119.875	2.926	0.004	**
Geographic distance	−0.223	0.090	116.651	−2.486	0.014	*

Signif. codes: 0

‘***’ 0.001 ‘**’ 0.01 ‘*’ 0.05 ‘.’ 0.1 ‘’ 1.

## 4. Discussion

In this study, we attempted to illuminate factors affecting cultural evolution of folk songs in the Ryukyu Archipelago by measuring melodic differences between songs, and evaluating the effects of variables that may have promoted or suppressed song diversification. To this end, we first classified songs into song groups, based on various sources of information, as aggregates of songs that have presumably originated from a common origin. We then quantified melodic differences between songs using Savage and Atkinson’s (2015) method, and examined whether a conventional phylogenetic approach supports our assumption of the shared origin of songs within a song group. Finally, we investigated effects of several variables on melodic differences between songs in the same song group to infer the factors affecting song diversification. The results of the analysis suggested the following: first, our classification of song groups is largely consistent with Neighbor-Net analysis (Huson & Bryant, 2006); second, a pair of songs tend to be more dissimilar when they are sung in different islands within the Ryukyu Archipelago than when sung in the same island, and when they are sung in different social contexts than when sung in the same social context; and third, the effects of geographic distance, recording year, or data source on song diversification are small or negligible.

In contrast to Nishikawa and Ihara (2022), who quantified distances between folk songs in the Ryukyu Archipelago on the basis of 26 CantoCore variables (Savage et al., 2012), this study focused strictly on the melodic variation (Savage & Atkinson, 2015), and observed clusters of songs in the Ryukyu scale, those in the ritsu scale, and those in the minyo scale in Neighbor-Net graphs. In addition, some songs in the Ryukyu scale of Okinawa formed a separate cluster, which may reflect the history that these songs were originally in the ritsu scale and were later modified to be in the Ryukyu scale. A limitation common to the present study and that of Nishikawa and Ihara (2022) is that their analyses did not take lyrics, an obviously important aspect of folk songs, into consideration. In fact, some songs of ‘Myakuni’, for example, were judged as close to each other despite their stark difference in lyrics. In other cases, songs with almost the same lyrics were judged as distant from each other for their melodic dissimilarity. These observations suggest that melodies and lyrics may be transmitted through different pathways. Folk songs in the Ryukyu Archipelago are sung in various local dialects of the Ryukyuan languages, which share a common ancestor with Japanese and diverged before the seventh century (Pellard, 2015). The Ryukyuan languages are classified into five groups – Amami, Okinawa, Miyako, Yaeyama, and Yonaguni – each corresponding to a region, and we focused on the four regions other than Yonaguni in this study. Lyrics of the folk songs are basically in the local dialects, although there are some exceptional cases in which songs are sung in different dialects (NHK, 1989–1993). It would be meaningful to examine whether the lyrics and melody of a song is always transmitted together, and whether the lyrics are more vulnerable to change than the melody is, particularly when the song diffuses into different regions.

The linear mixed model analysis suggested that a pair of melodies within the same song group tend to be more dissimilar when they are recorded in different islands than when recorded in the same islands, and when they are sung in different social contexts than when sung in the same social context. The reason why two sister songs separated by the sea tend to be more dissimilar may be that travel is more difficult and interactions less frequent between than within islands. Our analysis further suggested that the key variable promoting diversification of two songs is whether one of them has crossed the ocean, but not the geographic distance between them, suggesting that the sea is a cultural barrier that is stronger than expected from mere distance. As for the effect of social context, our results suggest that sister songs tend to be more dissimilar when one of them comes to be sung in a social context that is different from the one in which they were originally sung. This is consistent with the finding in a previous study that musical characteristics differ depending on their context (Mehr et al., 2019), suggesting the importance of music’s social function. Nishikawa and Ihara (2022) also found that songs sung in work-related contexts (i.e. work songs) tend to vary more between regions than child, ritual, or amusement songs. In contrast, the effect of difference in recording years was not significant in any cases. Although the difference in recording years between melodies was 52 years at maximum, this may be too short for any changes in the melodies of folk songs to be detected, considering the fact that it is getting easier to listen to and imitate older performances of folk songs because of the recent developments in recording technology, as Pendlebury (2020) pointed out. A caveat to our interpretation of the LMM results is that the model does not include the divergence time between pairs of melodies as an independent variable; thus, it could be that pairs of sister songs sung in different islands or different social contexts tend to be dissimilar because they tend to have diverged earlier than other pairs of sister songs, a possibility that seems to us unlikely, but cannot be tested with the current data. Note that whereas melodies in some song groups might change more rapidly or slowly than those in other song groups depending on, for example, the number of notes with strong rhythmic functions, the resulting variance in melodic similarity between song groups is taken into consideration by the incorporation of the random effect of song groups.

In conclusion, our quantitative analysis on folk songs in the Ryukyu Archipelago suggests that diversification of sister songs derived from a common origin is promoted when one of them is transmitted from one island to another, and when one of them undergoes a change of the social context in which it is sung. Our conclusion is based only on the difference in melody between songs, and future studies should also consider other aspects of song variation, most notably the difference in lyrics between songs, to further explore the factors affecting cultural evolution of folk songs.

## Supporting information

Nishikawa and Ihara supplementary material 1 Nishikawa and Ihara supplementary material

Nishikawa and Ihara supplementary material 2 Nishikawa and Ihara supplementary material

## Supplementary material

The supplementary material for this article can be found at https://doi.org/10.1017/ehs.2025.10010.

## Research transparency and reproducibility

Song data are available from Supplementary material. R source code to calculate PID between melodies is available at https://github.com/pesavage/melodic-evolution.
