# Supplementary material for: Exploring factors for melodic diversification of folk songs in the Ryukyu Archipelago
Source: Evol Hum Sci. 2025 Jul 22;7:e23. doi: 10.1017/ehs.2025.10010 (PMC12516598; doi:10.1017/ehs.2025.10010)
Supplement: Nishikawa and Ihara supplementary material 2 — Nishikawa and Ihara supplementary material [file S2513843X25100108sup002.docx]

**Supplementary Table S2.** Results of model selection for all melodies. Model selection with standardised variables and (a) parameter set 1 and (b) parameter set 2. Model selection with standardised variables and (c) parameter set 1 and (d) parameter set 2 made to include geographic distance and difference in recording years. Model selection with non-standardised variables and (e) parameter set 1 and (f) parameter set 2. Model selection with non-standardised variables and (g) parameter set 1 and (h) parameter set 2 made to include difference in recording years.

| (a) |  |  |  |  |  |  |  |  |  | (b) |  |  |  |  |  |  |  |  |
| --- | --- | --- | --- | --- | --- | --- | --- | --- | --- | --- | --- | --- | --- | --- | --- | --- | --- | --- |
| Random |  |  |  |  |  |  |  |  |  | Random |  |  |  |  |  |  |  |  |
|  | Eliminated | npar | logLik | AIC | LRT | Df | Pr(>Chisq) |  |  |  | Eliminated | npar | logLik | AIC | LRT | Df | Pr(>Chisq) |  |
| <none> | NA | 8 | -359.815 | 735.630 | NA | NA | NA |  |  | <none> | NA | 8 | -356.656 | 729.312 | NA | NA | NA |  |
| Random effects | 0 | 7 | -410.655 | 835.311 | 101.681 | 1 | 6.52E-24 | *** |  | Random effects | 0 | 7 | -409.882 | 833.764 | 106.452 | 1 | 5.87E-25 | *** |
| Fixed |  |  |  |  |  |  |  |  |  | Fixed |  |  |  |  |  |  |  |  |
|  | Eliminated | Sum Sq | Mean Sq | NumDF | DenDF | F value | Pr(>F) |  |  |  | Eliminated | Sum Sq | Mean Sq | NumDF | DenDF | F value | Pr(>F) |  |
| Geographic distance | 1 | 0.074 | 0.074 | 1 | 294.674 | 0.162 | 0.688 |  |  | Source of melodies | 1 | 0.531 | 0.531 | 1 | 301.523 | 1.186 | 0.277 |  |
| Source of melodies | 2 | 0.652 | 0.652 | 1 | 302.907 | 1.424 | 0.234 |  |  | Geographic distance | 2 | 0.809 | 0.809 | 1 | 295.038 | 1.804 | 0.180 |  |
| Year | 3 | 0.370 | 0.370 | 1 | 281.880 | 0.805 | 0.370 |  |  | Year | 3 | 1.383 | 1.383 | 1 | 281.402 | 3.072 | 0.081 | . |
| Island | 0 | 3.620 | 3.620 | 1 | 296.831 | 7.878 | 0.005 | ** |  | Island | 0 | 2.892 | 2.892 | 1 | 296.019 | 6.376 | 0.012 | * |
| Social context | 0 | 11.884 | 11.884 | 1 | 300.836 | 25.859 | 6.47E-07 | *** |  | Social context | 0 | 11.950 | 11.950 | 1 | 302.123 | 26.344 | 5.12E-07 | *** |
|  |  |  |  |  |  |  |  |  |  |  |  |  |  |  |  |  |  |  |
| (c) |  |  |  |  |  |  |  |  |  | (d) |  |  |  |  |  |  |  |  |
| Random |  |  |  |  |  |  |  |  |  | Random |  |  |  |  |  |  |  |  |
|  | Eliminated | npar | logLik | AIC | LRT | Df | Pr(>Chisq) |  |  |  | Eliminated | npar | logLik | AIC | LRT | Df | Pr(>Chisq) |  |
| <none> | NA | 7 | -359.879 | 733.757 | NA | NA | NA |  |  | <none> | NA | 7 | -356.620 | 727.240 | NA | NA | NA |  |
| Random effects | 0 | 6 | -410.468 | 832.937 | 101.179 | 1 | 8.40E-24 | *** |  | Random effects | 0 | 6 | -411.171 | 834.342 | 109.102 | 1 | 1.54E-25 | *** |
| Fixed |  |  |  |  |  |  |  |  |  | Fixed |  |  |  |  |  |  |  |  |
|  | Eliminated | Sum Sq | Mean Sq | NumDF | DenDF | F value | Pr(>F) |  |  |  | Eliminated | Sum Sq | Mean Sq | NumDF | DenDF | F value | Pr(>F) |  |
| Island | 0 | 3.356 | 3.356 | 1 | 298.090 | 7.282 | 0.007 | ** |  | Island | 0 | 4.103 | 4.103 | 1 | 296.967 | 9.151 | 0.003 | ** |
| Geographic distance | 0 | 0.097 | 0.097 | 1 | 296.151 | 0.210 | 0.647 |  |  | Geographic distance | 0 | 0.809 | 0.809 | 1 | 295.038 | 1.804 | 0.180 |  |
| Year | 0 | 0.424 | 0.424 | 1 | 279.046 | 0.920 | 0.338 |  |  | Year | 0 | 1.708 | 1.708 | 1 | 278.335 | 3.809 | 0.052 | . |
| Social context | 0 | 11.945 | 11.945 | 1 | 299.610 | 25.920 | 6.30E-07 | *** |  | Social context | 0 | 12.168 | 12.168 | 1 | 301.246 | 27.137 | 3.52E-07 | *** |
|  |  |  |  |  |  |  |  |  |  |  |  |  |  |  |  |  |  |  |
| (e) |  |  |  |  |  |  |  |  |  | (f) |  |  |  |  |  |  |  |  |
| Random |  |  |  |  |  |  |  |  |  | Random |  |  |  |  |  |  |  |  |
|  | Eliminated | npar | logLik | AIC | LRT | Df | Pr(>Chisq) |  |  |  | Eliminated | npar | logLik | AIC | LRT | Df | Pr(>Chisq) |  |
| <none> | NA | 7 | 200.423 | -386.846 | NA | NA | NA |  |  | <none> | NA | 7 | 244.614 | -475.227 | NA | NA | NA |  |
| Random effects | 0 | 6 | 149.833 | -287.667 | 101.179 | 1 | 8.40E-24 | *** |  | Random effects | 0 | 6 | 190.063 | -368.125 | 109.102 | 1 | 1.54E-25 | *** |
| Fixed |  |  |  |  |  |  |  |  |  | Fixed |  |  |  |  |  |  |  |  |
|  | Eliminated | Sum Sq | Mean Sq | NumDF | DenDF | F value | Pr(>F) |  |  |  | Eliminated | Sum Sq | Mean Sq | NumDF | DenDF | F value | Pr(>F) |  |
| Geographic distance | 1 | 0.002 | 0.002 | 1 | 296.151 | 0.210 | 0.647 |  |  | Geographic distance | 1 | 0.015 | 0.015 | 1 | 295.038 | 1.804 | 0.180 |  |
| Year | 2 | 0.009 | 0.009 | 1 | 281.880 | 0.805 | 0.370 |  |  | Year | 2 | 0.026 | 0.026 | 1 | 281.402 | 3.072 | 0.081 | . |
| Island | 0 | 0.090 | 0.090 | 1 | 296.831 | 7.878 | 0.005 | ** |  | Island | 0 | 0.055 | 0.055 | 1 | 296.019 | 6.376 | 0.012 | * |
| Social context | 0 | 0.294 | 0.294 | 1 | 300.836 | 25.859 | 6.47E-07 | *** |  | Social context | 0 | 0.226 | 0.226 | 1 | 302.123 | 26.344 | 5.12E-07 | *** |
|  |  |  |  |  |  |  |  |  |  |  |  |  |  |  |  |  |  |  |
| (g) |  |  |  |  |  |  |  |  |  | (h) |  |  |  |  |  |  |  |  |
| Random |  |  |  |  |  |  |  |  |  | Random |  |  |  |  |  |  |  |  |
|  | Eliminated | npar | logLik | AIC | LRT | Df | Pr(>Chisq) |  |  |  | Eliminated | npar | logLik | AIC | LRT | Df | Pr(>Chisq) |  |
| <none> | NA | 7 | 200.423 | -386.846 | NA | NA | NA |  |  | <none> | NA | 7 | 244.614 | -475.227 | NA | NA | NA |  |
| Random effects | 0 | 6 | 149.833 | -287.667 | 101.179 | 1 | 8.40E-24 | *** |  | Random effects | 0 | 6 | 190.063 | -368.125 | 109.102 | 1 | 1.54E-25 | *** |
| Fixed |  |  |  |  |  |  |  |  |  | Fixed |  |  |  |  |  |  |  |  |
|  | Eliminated | Sum Sq | Mean Sq | NumDF | DenDF | F value | Pr(>F) |  |  |  | Eliminated | Sum Sq | Mean Sq | NumDF | DenDF | F value | Pr(>F) |  |
| Geographic distance | 1 | 0.002 | 0.002 | 1 | 296.151 | 0.210 | 0.647 |  |  | Geographic distance | 1 | 0.015 | 0.015 | 1 | 295.038 | 1.804 | 0.180 |  |
| Island | 0 | 0.095 | 0.095 | 1 | 295.484 | 8.366 | 0.004 | ** |  | Island | 0 | 0.063 | 0.063 | 1 | 294.746 | 7.447 | 0.007 | ** |
| Year | 0 | 0.009 | 0.009 | 1 | 281.880 | 0.805 | 0.370 |  |  | Year | 0 | 0.026 | 0.026 | 1 | 281.402 | 3.072 | 0.081 | . |
| Social context | 0 | 0.293 | 0.293 | 1 | 300.163 | 25.786 | 6.71E-07 | *** |  | Social context | 0 | 0.226 | 0.226 | 1 | 301.361 | 26.479 | 4.81E-07 | *** |
|  |  |  |  |  |  |  |  |  |  |  |  |  |  |  |  |  |  |  |
| --- |  |  |  |  |  |  |  |  |  |  |  |  |  |  |  |  |  |  |
| Signif. codes: 0 ‘***’ 0.001 ‘**’ 0.01 ‘*’ 0.05 ‘.’ 0.1 ‘ ’ 1 | | | |  |  |  |  |  |  |  |  |  |  |  |  |  |  |  |

**Supplementary Table S3.** Results of model selection for melodies from SJF. Model selection with standardised variables and (a) parameter set 1 and (b) parameter set 2. Model selection with standardised variables and (c) parameter set 1 and (d) parameter set 2 made not to include difference in islands. Model selection with standardised variables and (e) parameter set 1 and (f) parameter set 2 made not to include geographic distance.

| (a) |  |  |  |  |  |  |  |  |  | (b) |  |  |  |  |  |  |  |  |
| --- | --- | --- | --- | --- | --- | --- | --- | --- | --- | --- | --- | --- | --- | --- | --- | --- | --- | --- |
| Random |  |  |  |  |  |  |  |  |  | Random |  |  |  |  |  |  |  |  |
|  | Eliminated | npar | logLik | AIC | LRT | Df | Pr(>Chisq) |  |  |  | Eliminated | npar | logLik | AIC | LRT | Df | Pr(>Chisq) |  |
| <none> | NA | 7 | -163.848 | 341.695 | NA | NA | NA |  |  | <none> | NA | 7 | -159.887 | 333.775 | NA | NA | NA |  |
| Random effects | 0 | 6 | -176.737 | 365.473 | 25.778 | 1 | 3.83E-07 | *** |  | Random effects | 0 | 6 | -178.119 | 368.239 | 36.464 | 1 | 1.56E-09 | *** |
| Fixed |  |  |  |  |  |  |  |  |  | Fixed |  |  |  |  |  |  |  |  |
|  | Eliminated | Sum Sq | Mean Sq | NumDF | DenDF | F value | Pr(>F) |  |  |  | Eliminated | Sum Sq | Mean Sq | NumDF | DenDF | F value | Pr(>F) |  |
| Year | 1 | 0.249 | 0.249 | 1 | 120.981 | 0.410 | 0.523 |  |  | Social context | 1 | 0.474 | 0.474 | 1 | 116.107 | 0.847 | 0.359 |  |
| Social context | 2 | 1.002 | 1.002 | 1 | 110.978 | 1.652 | 0.201 |  |  | Year | 2 | 0.881 | 0.881 | 1 | 121.190 | 1.596 | 0.209 |  |
| Island | 0 | 6.361 | 6.361 | 1 | 120.473 | 10.635 | 0.001 | ** |  | Island | 0 | 4.767 | 4.767 | 1 | 119.875 | 8.564 | 0.004 | ** |
| Geographic distance | 0 | 4.805 | 4.805 | 1 | 117.075 | 8.034 | 0.005 | ** |  | Geographic distance | 0 | 3.439 | 3.439 | 1 | 116.651 | 6.179 | 0.014 | * |
|  |  |  |  |  |  |  |  |  |  |  |  |  |  |  |  |  |  |  |
| (c) |  |  |  |  |  |  |  |  |  | (d) |  |  |  |  |  |  |  |  |
| Random |  |  |  |  |  |  |  |  |  | Random |  |  |  |  |  |  |  |  |
|  | Eliminated | npar | logLik | AIC | LRT | Df | Pr(>Chisq) |  |  |  | Eliminated | npar | logLik | AIC | LRT | Df | Pr(>Chisq) |  |
| <none> | NA | 6 | -167.662 | 347.324 | NA | NA | NA |  |  | <none> | NA | 6 | -162.613 | 337.226 | NA | NA | NA |  |
| Random effects | 0 | 5 | -182.065 | 374.131 | 28.806 | 1 | 8.00E-08 | *** |  | Random effects | 0 | 5 | -181.385 | 372.770 | 37.544 | 1 | 8.94E-10 | *** |
| Fixed |  |  |  |  |  |  |  |  |  | Fixed |  |  |  |  |  |  |  |  |
|  | Eliminated | Sum Sq | Mean Sq | NumDF | DenDF | F value | Pr(>F) |  |  |  | Eliminated | Sum Sq | Mean Sq | NumDF | DenDF | F value | Pr(>F) |  |
| Year | 1 | 0.348 | 0.348 | 1 | 121.999 | 0.529 | 0.469 |  |  | Social context | 1 | 0.353 | 0.353 | 1 | 113.734 | 0.589 | 0.445 |  |
| Geographic distance | 2 | 0.603 | 0.603 | 1 | 118.183 | 0.915 | 0.341 |  |  | Geographic distance | 2 | 0.468 | 0.468 | 1 | 116.383 | 0.790 | 0.376 |  |
| Social context | 3 | 0.966 | 0.966 | 1 | 108.587 | 1.457 | 0.230 |  |  | Year | 3 | 1.047 | 1.047 | 1 | 123.746 | 1.766 | 0.186 |  |
|  |  |  |  |  |  |  |  |  |  |  |  |  |  |  |  |  |  |  |
| (e) |  |  |  |  |  |  |  |  |  | (f) |  |  |  |  |  |  |  |  |
| Random |  |  |  |  |  |  |  |  |  | Random |  |  |  |  |  |  |  |  |
|  | Eliminated | npar | logLik | AIC | LRT | Df | Pr(>Chisq) |  |  |  | Eliminated | npar | logLik | AIC | LRT | Df | Pr(>Chisq) |  |
| <none> | NA | 6 | -165.807 | 343.614 | NA | NA | NA |  |  | <none> | NA | 6 | -161.004 | 334.008 | NA | NA | NA |  |
| Random effects | 0 | 5 | -176.416 | 362.832 | 21.217 | 1 | 4.10E-06 | *** |  | Random effects | 0 | 5 | -177.060 | 364.121 | 32.113 | 1 | 1.45E-08 | *** |
| Fixed |  |  |  |  |  |  |  |  |  | Fixed |  |  |  |  |  |  |  |  |
|  | Eliminated | Sum Sq | Mean Sq | NumDF | DenDF | F value | Pr(>F) |  |  |  | Eliminated | Sum Sq | Mean Sq | NumDF | DenDF | F value | Pr(>F) |  |
| Year | 1 | 0.307 | 0.307 | 1 | 121.626 | 0.472 | 0.493 |  |  | Year | 1 | 0.729 | 0.729 | 1 | 121.980 | 1.237 | 0.268 |  |
| Social context | 2 | 1.538 | 1.538 | 1 | 105.007 | 2.368 | 0.127 |  |  | Social context | 2 | 1.176 | 1.176 | 1 | 111.108 | 1.974 | 0.163 |  |
| Island | 3 | 2.480 | 2.480 | 1 | 122.036 | 3.854 | 0.052 | . |  | Island | 3 | 1.903 | 1.903 | 1 | 121.295 | 3.230 | 0.075 | . |
|  |  |  |  |  |  |  |  |  |  |  |  |  |  |  |  |  |  |  |
| --- |  |  |  |  |  |  |  |  |  |  |  |  |  |  |  |  |  |  |
| Signif. codes: 0 ‘***’ 0.001 ‘**’ 0.01 ‘*’ 0.05 ‘.’ 0.1 ‘ ’ 1 | | | |  |  |  |  |  |  |  |  |  |  |  |  |  |  |  |

**Supplementary results of linear mixed model**

**Supplementary Table S4.** Random effect of LMM analysis for all melodies. Model (2) with standardised variables and parameter set (a) 1 or (b) 2.

| (a) | | | |
| --- | --- | --- | --- |
| Groups | Name | Variance | Std.Dev. |
| Song group | (Intercept) | 0.5024 | 0.7088 |
| Residual |  | 0.4596 | 0.6779 |
|  |  |  |  |
| (b) | | | |
| Groups | Name | Variance | Std.Dev. |
| Song group | (Intercept) | 0.5185 | 0.7200 |
| Residual |  | 0.4536 | 0.6735 |

**Supplementary Table S5.** Results of LMM analysis for all melodies. Model (2) with standardised variables and parameter set 1.

|  | Estimate | Std. Error | df | t value | Pr(>\|t\|) |  |
| --- | --- | --- | --- | --- | --- | --- |
| (Intercept) | -0.334 | 0.131 | 35.402 | -2.539 | 0.016 | * |
| Island | 0.137 | 0.049 | 296.831 | 2.807 | 0.005 | ** |
| Social context | 0.276 | 0.054 | 300.836 | 5.085 | 6.47E-07 | *** |
| --- |  |  |  |  |  |  |
| Signif. codes: 0 ‘***’ 0.001 ‘**’ 0.01 ‘*’ 0.05 ‘.’ 0.1 ‘ ’ 1 | | | | | | |

**Supplementary Table S6.** Results of LMM analysis for all melodies. Model (2) with non-standardised variables and parameter set 1.

|  | Estimate | Std. Error | df | t value | Pr(>\|t\|) |  |
| --- | --- | --- | --- | --- | --- | --- |
| (Intercept) | 0.382 | 0.021 | 38.556 | 18.118 | 1.85E-20 | *** |
| Island | 0.044 | 0.016 | 296.831 | 2.807 | 0.005 | ** |
| Social context | 0.164 | 0.032 | 300.836 | 5.085 | 6.47E-07 | *** |
| --- |  |  |  |  |  |  |
| Signif. codes: 0 ‘***’ 0.001 ‘**’ 0.01 ‘*’ 0.05 ‘.’ 0.1 ‘ ’ 1 | | | | | | |

**Supplementary Table S7.** Results of LMM analysis for melodies from SJF. Model (3) with standardised variables and parameter set 1.

|  | Estimate | Std. Error | df | t value | Pr(>\|t\|) |  |
| --- | --- | --- | --- | --- | --- | --- |
| (Intercept) | -0.020 | 0.277 | 11.131 | -0.074 | 0.942 |  |
| Island | 0.324 | 0.099 | 120.473 | 3.261 | 0.001 | ** |
| Geographic distance | -0.263 | 0.093 | 117.075 | -2.835 | 0.005 | ** |
| --- |  |  |  |  |  |  |
| Signif. codes: 0 ‘***’ 0.001 ‘**’ 0.01 ‘*’ 0.05 ‘.’ 0.1 ‘ ’ 1 | | | | | | |

To further examine possible effects of geographic distance and difference in recording years, variables not included in model (2), model selection was performed with forced entry of $D$ and $T$, using standardised variables (Supplementary Table S2cd). The following model was selected for both parameter sets:

$$Y_{ij}=\beta_{0}+\beta_{1}I_{ij}+\beta_{2}D_{ij}+\beta_{3}T_{ij}+\beta_{4}C_{ij}+r_{0k}. (4)$$

The estimated partial regression coefficients are shown in Supplementary Table S8. While the effect of difference in islands was significant as in model (2), a significant effect was not found for geographic distance or difference in recording years. Although $I$ and $D$ were positively correlated (*r* = 0.404, *p* < 0.01), no multicollinearity was observed in model (4) (Supplementary Table S12).

**Supplementary Table S8.** Results of LMM analysis for all melodies. Model (4) with standardised variables and parameter set (a) 1 or (b) 2.

| (a) |  |  |  |  |  |  |
| --- | --- | --- | --- | --- | --- | --- |
|  | Estimate | Std. Error | df | t value | Pr(>\|t\|) |  |
| (Intercept) | -0.329 | 0.132 | 35.505 | -2.493 | 0.017 | * |
| Island | 0.157 | 0.058 | 298.090 | 2.698 | 0.007 | ** |
| Geographic distance | -0.023 | 0.051 | 296.151 | -0.458 | 0.647 |  |
| Year | 0.041 | 0.042 | 279.046 | 0.959 | 0.338 |  |
| Social context | 0.277 | 0.054 | 299.610 | 5.091 | 6.30E-07 | *** |
|  |  |  |  |  |  |  |
| (b) |  |  |  |  |  |  |
|  | Estimate | Std. Error | df | t value | Pr(>\|t\|) |  |
| (Intercept) | -0.273 | 0.133 | 35.286 | -2.042 | 0.049 | * |
| Island | 0.173 | 0.057 | 296.967 | 3.025 | 0.003 | ** |
| Geographic distance | -0.068 | 0.050 | 295.038 | -1.343 | 0.180 |  |
| Year | 0.081 | 0.042 | 278.335 | 1.952 | 0.052 | . |
| Social context | 0.281 | 0.054 | 301.246 | 5.209 | 3.52E-07 | *** |
|  |  |  |  |  |  |  |
| --- |  |  |  |  |  |  |
| Signif. codes: 0 ‘***’ 0.001 ‘**’ 0.01 ‘*’ 0.05 ‘.’ 0.1 ‘ ’ 1 | | | | | | |

As an attempt to quantify the effect of unit increase of geographic distance on song dissimilarity, we replaced $I$ with $D$ in model (2) to obtain

$$Y_{ij}=\beta_{0}+\beta_{2}D_{ij}+\beta_{4}C_{ij}+r_{0k}, (5)$$

for which partial regression coefficients were estimated using non-standardised variables (Supplementary Table S9). The effect of geographic distance was not significant but slightly positive, with estimates of 4.20×10^-4^ for parameter set 1 and 1.22×10^-4^ for parameter set 2, which may be interpreted as the increase of distance between a pair of melodies in the same song group when their recording sites get 1 km more distant from each other. These were quite small considering the actual geographic distance between recording sites and relative to the effect of the difference in islands in the previous model.

**Supplementary Table S9.** Results of LMM analysis for all melodies. Model (5) with non-standardised variables and parameter set (a) 1 or (b) 2.

| (a) |  |  |  |  |  |  |
| --- | --- | --- | --- | --- | --- | --- |
|  | Estimate | Std. Error | df | t value | Pr(>\|t\|) |  |
| (Intercept) | 0.384 | 0.023 | 46.352 | 16.689 | 3.16E-21 | *** |
| Geographic distance | 4.20.E-04 | 3.68.E-04 | 293.832 | 1.141 | 0.255 |  |
| Social context | 0.156 | 0.033 | 303.361 | 4.785 | 2.67E-06 | *** |
|  |  |  |  |  |  |  |
| (b) |  |  |  |  |  |  |
|  | Estimate | Std. Error | df | t value | Pr(>\|t\|) |  |
| (Intercept) | 0.312 | 0.020 | 45.311 | 15.497 | 9.94E-20 | *** |
| Geographic distance | 1.22.E-04 | 3.20.E-04 | 293.191 | 0.381 | 0.704 |  |
| Social context | 0.139 | 0.028 | 303.804 | 4.885 | 1.67E-06 | *** |
|  |  |  |  |  |  |  |
| --- |  |  |  |  |  |  |
| Signif. codes: 0 ‘***’ 0.001 ‘**’ 0.01 ‘*’ 0.05 ‘.’ 0.1 ‘ ’ 1 | | | | | | |

Model selection was also made with forced entry of $T$, the effect of difference in recording years, using non-standardised variables (Supplementary Table S2gh). The following model was selected for both parameter sets:

$$Y_{ij}=\beta_{0}+\beta_{1}I_{ij}+\beta_{3}T_{ij}+\beta_{4}C_{ij}+r_{0k}. (6)$$

The estimates of partial regression coefficients are shown in Supplementary Table S10. The effect of difference in recording years was not significant, with estimates of 3.19×10^-4^ for parameter set 1 and 5.39×10^-4^ for parameter set 2. These may be interpreted as the increase of distance between a pair of melodies in the same song group for one-year increase of the difference in their recording years, which turned out to be very small.

**Supplementary Table S10.** Results of LMM analysis for all melodies. Model (6) with non-standardised variables and parameter set (a) 1 or (b) 2.

| (a) |  |  |  |  |  |  |
| --- | --- | --- | --- | --- | --- | --- |
|  | Estimate | Std. Error | df | t value | Pr(>\|t\|) |  |
| (Intercept) | 0.376 | 0.022 | 47.309 | 16.894 | 1.14E-21 | *** |
| Island | 0.046 | 0.016 | 295.484 | 2.892 | 0.004 | ** |
| Year | 3.19E-04 | 3.55E-04 | 281.880 | 0.897 | 0.370 |  |
| Social context | 0.164 | 0.032 | 300.163 | 5.078 | 6.71E-07 | *** |
|  |  |  |  |  |  |  |
| (b) |  |  |  |  |  |  |
|  | Estimate | Std. Error | df | t value | Pr(>\|t\|) |  |
| (Intercept) | 0.296 | 0.020 | 46.790 | 15.159 | 1.12E-19 | *** |
| Island | 0.037 | 0.014 | 294.746 | 2.729 | 0.007 | ** |
| Year | 5.39E-04 | 3.08E-04 | 281.402 | 1.753 | 0.081 | . |
| Social context | 0.144 | 0.028 | 301.361 | 5.146 | 4.81E-07 | *** |
|  |  |  |  |  |  |  |
| --- |  |  |  |  |  |  |
| Signif. codes: 0 ‘***’ 0.001 ‘**’ 0.01 ‘*’ 0.05 ‘.’ 0.1 ‘ ’ 1 | | | | | | |

We conducted additional analyses using only melodies from SJF (126 pairs of melodies from the same song group). When model selection was made under the restriction that at most one of $I$ and $D$ is entered into the model, a model without any fixed effect,

$$Y_{ij}=\beta_{0}+r_{0k}, (7)$$

was selected for both parameter sets (Supplementary Table S3c-f). The estimated partial correlation coefficients are shown in Supplementary Table S11.

**Supplementary Table S11.** Results of LMM analysis for melodies from SJF. Model (7) with standardised variables and parameter set (a) 1 or (b) 2.

| (a) |  |  |  |  |  |  |
| --- | --- | --- | --- | --- | --- | --- |
|  | Estimate | Std. Error | df | t value | Pr(>\|t\|) |  |
| (Intercept) | -0.097 | 0.263 | 10.819 | -0.368 | 0.720 |  |
|  |  |  |  |  |  |  |
| (b) |  |  |  |  |  |  |
|  | Estimate | Std. Error | df | t value | Pr(>\|t\|) |  |
| (Intercept) | -0.088 | 0.264 | 11.098 | -0.332 | 0.746 |  |
|  |  |  |  |  |  |  |
| --- |  |  |  |  |  |  |
| Signif. codes: 0 ‘***’ 0.001 ‘**’ 0.01 ‘*’ 0.05 ‘.’ 0.1 ‘ ’ 1 | | | | | | |

**Supplementary Table S12.** Results of checking multicollinearity in the model (3) with (a) the parameter set 1 and (b) the parameter set 2. All VIFs were sufficiently small.

| (a) |  |  |  |  |  |
| --- | --- | --- | --- | --- | --- |
| Term | VIF | VIF 95% CI | Increased SE | Tolerance | Tolerance 95% CI |
| *I* | 1.09 | [1.02, 1.38] | 1.04 | 0.92 | [0.73, 0.98] |
| *D* | 1.06 | [1.01, 1.44] | 1.03 | 0.94 | [0.69, 0.99] |
| *T* | 1.03 | [1.00, 2.61] | 1.01 | 0.97 | [0.38, 1.00] |
| *C* | 1.01 | [1.00, 1.59e+07] | 1.00 | 1.00 | [0.00, 1.00] |
|  |  |  |  |  |  |
| (b) |  |  |  |  |  |
| Term | VIF | VIF 95% CI | Increased SE | Tolerance | Tolerance 95% CI |
| *I* | 1.08 | [1.02, 1.38] | 1.04 | 0.92 | [0.72, 0.98] |
| *D* | 1.06 | [1.01, 1.47] | 1.03 | 0.94 | [0.68, 0.99] |
| *T* | 1.03 | [1.00, 2.80] | 1.01 | 0.97 | [0.36, 1.00] |
| *C* | 1.00 | [1.00, 3.67e+08] | 1.00 | 1.00 | [0.00, 1.00] |

**Supplementary Table S13.** Results of checking multicollinearity in the model (6) when limited to the melodies from SJF with (a) the parameter set 1 and (b) the parameter set 2. All VIFs were sufficiently small.

| (a) |  |  |  |  |  |
| --- | --- | --- | --- | --- | --- |
| Term | VIF | VIF 95% CI | Increased SE | Tolerance | Tolerance 95% CI |
| *I* | 1.47 | [1.24, 1.94] | 1.21 | 0.68 | [0.51, 0.81] |
| *D* | 1.47 | [1.24, 1.94] | 1.21 | 0.68 | [0.51, 0.81] |
|  |  |  |  |  |  |
| (b) |  |  |  |  |  |
| Term | VIF | VIF 95% CI | Increased SE | Tolerance | Tolerance 95% CI |
| *I* | 1.47 | [1.25, 1.94] | 1.21 | 0.68 | [0.51, 0.81] |
| *D* | 1.47 | [1.25, 1.94] | 1.21 | 0.68 | [0.51, 0.81] |


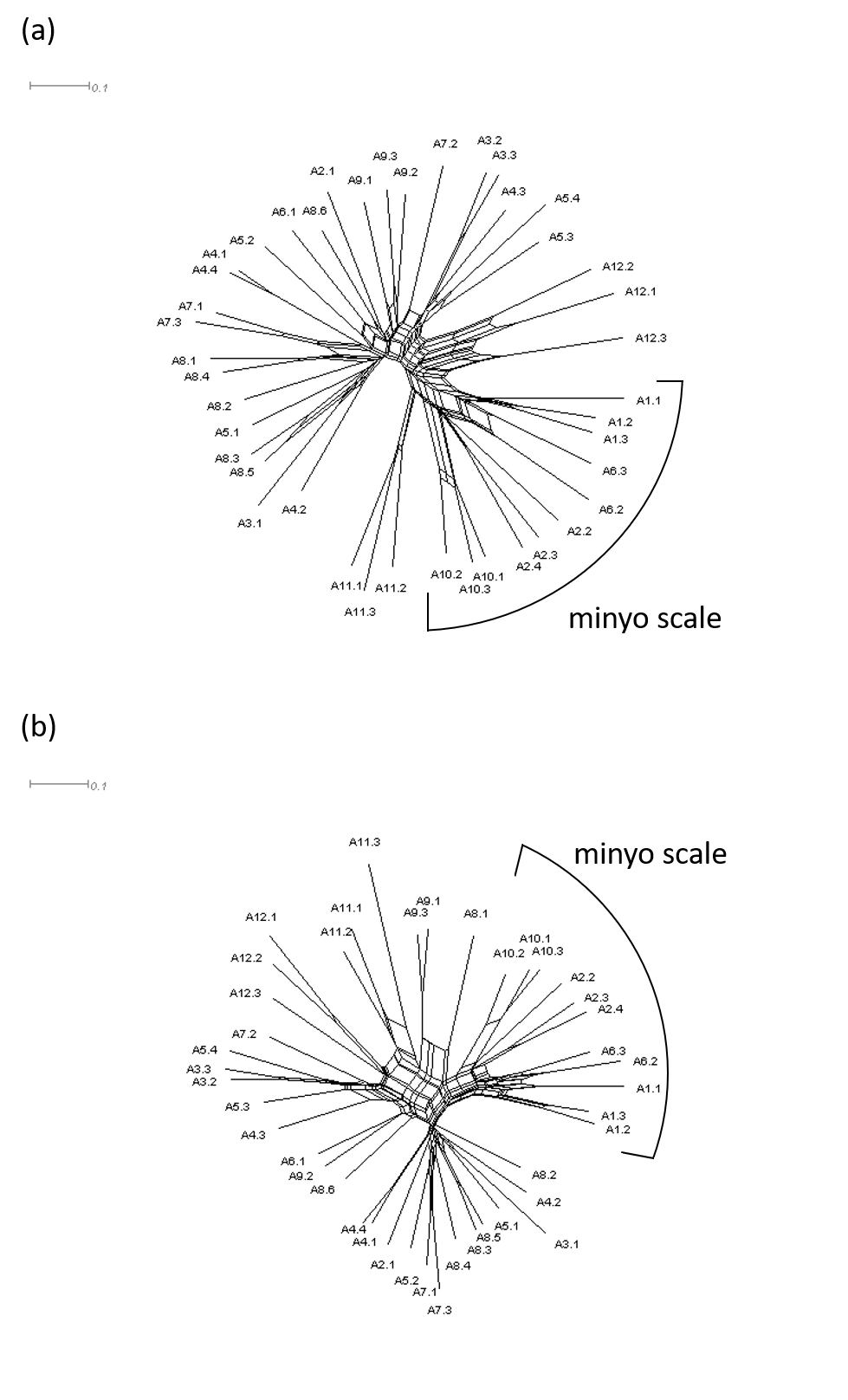


**Supplementary Figure S1.** Neighbor-Net graphs based on the distances between melodies of Amami with (a) the parameter set 1 (*δ* = 0.3812) and (b) the parameter set 2 (*δ* = 0.353).


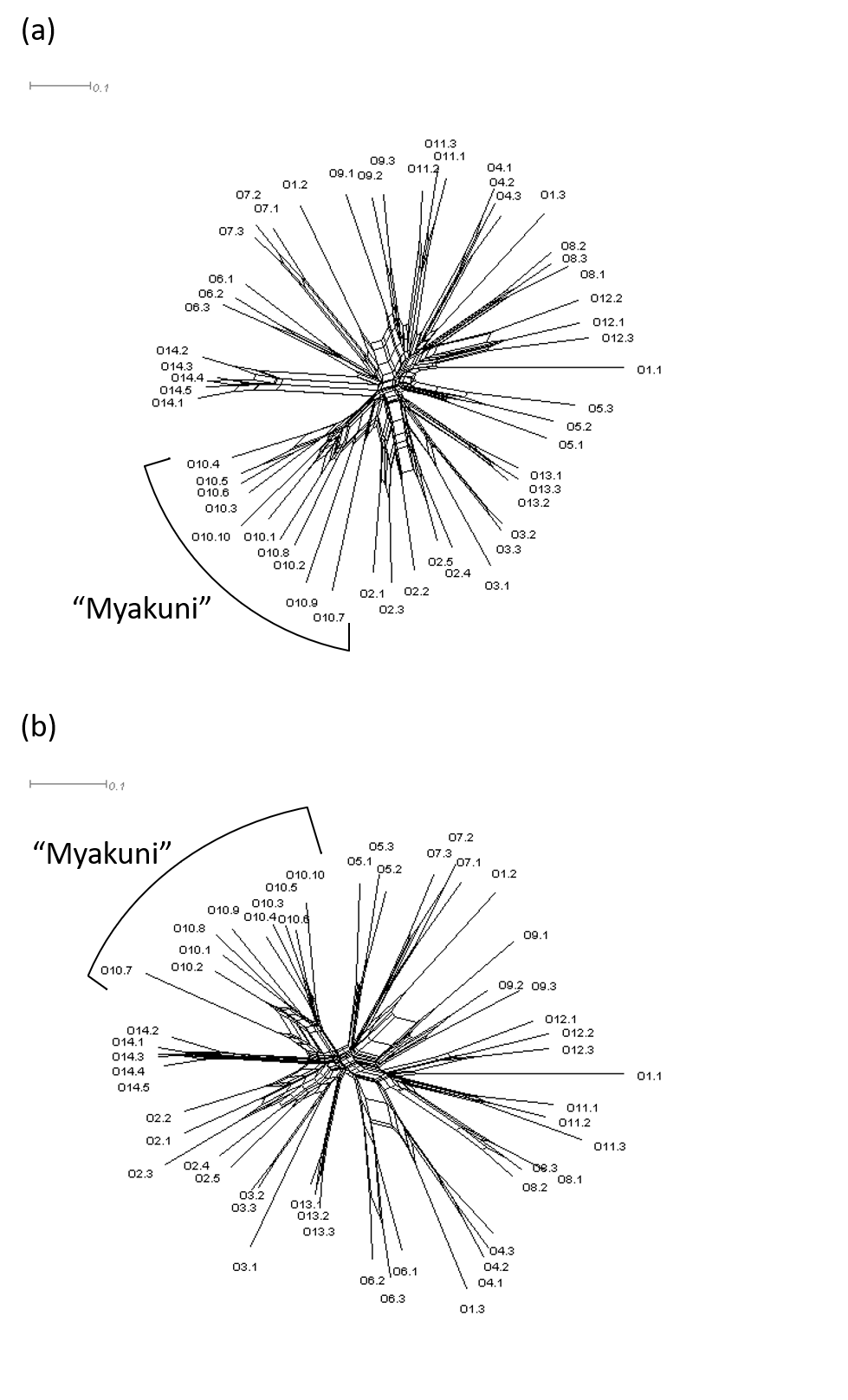


**Supplementary Figure S2.** Neighbor-Net graphs based on the distances between melodies of Okinawa with (a) the parameter set 1 (*δ* = 0.4167) and (b) the parameter set 2 (*δ* = 0.3875).


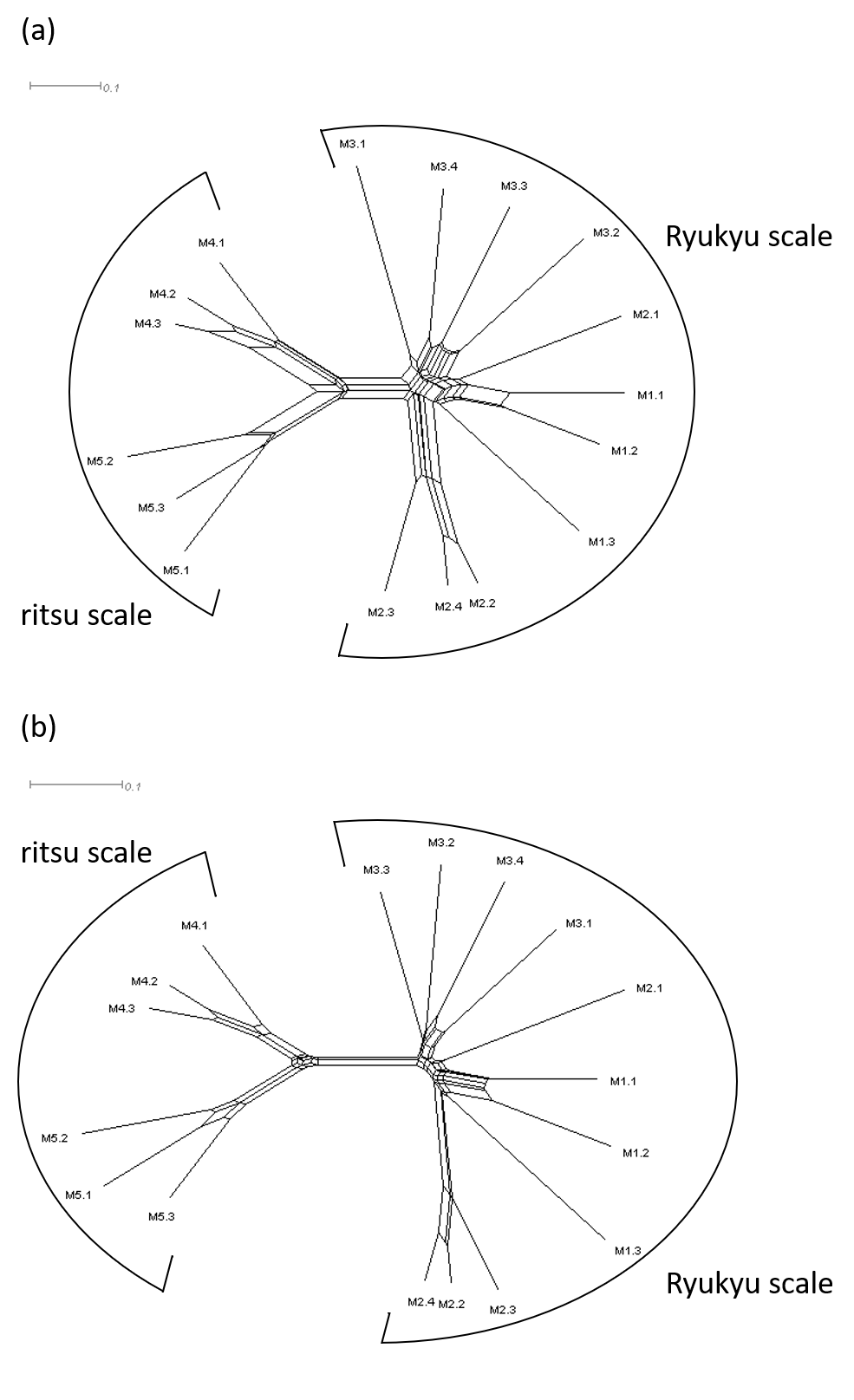


**Supplementary Figure S3.** Neighbor-Net graphs based on the distances between melodies of Miyako with (a) the parameter set 1 (*δ* = 0.3042) and (b) the parameter set 2 (*δ* = 0.2799).


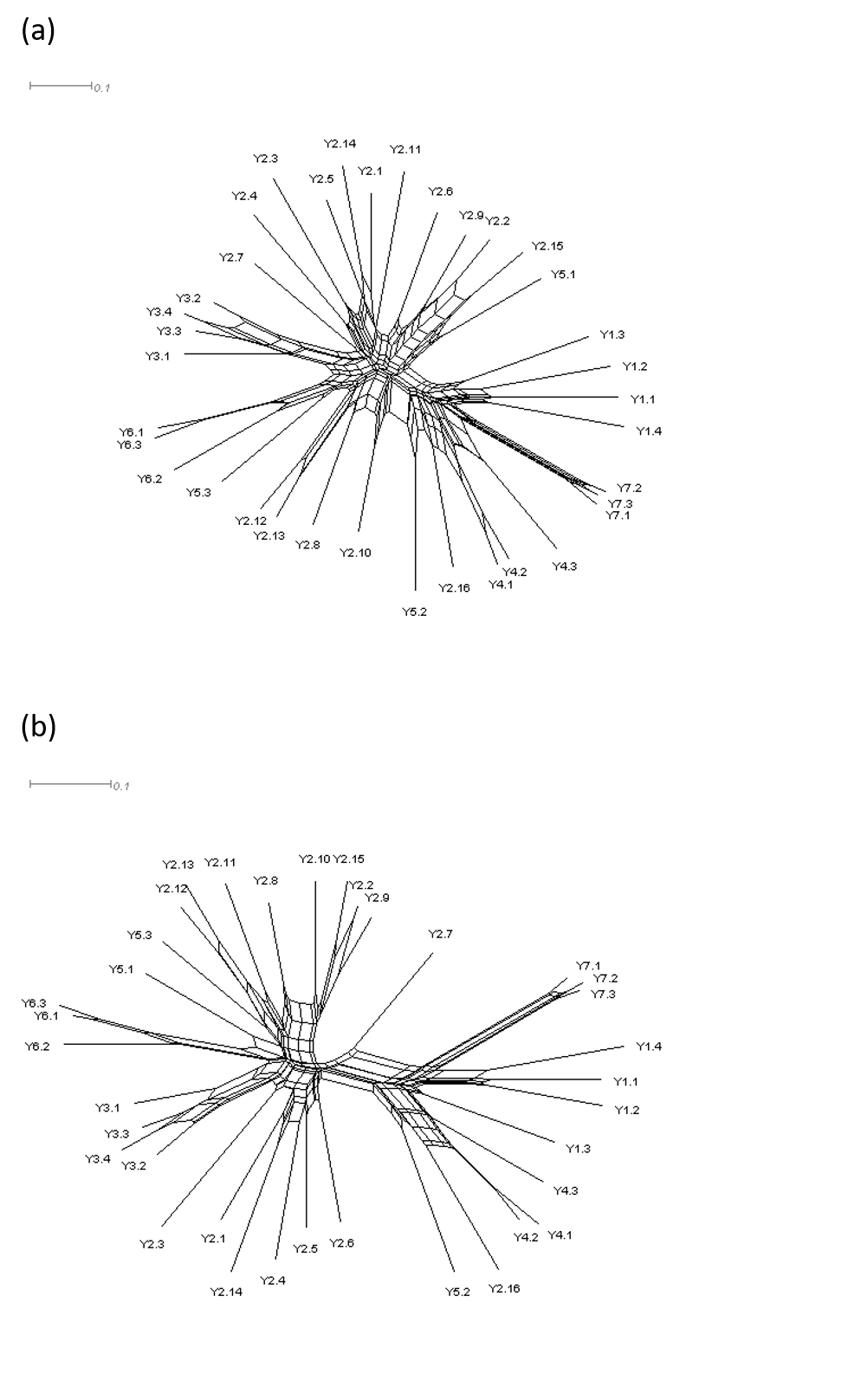


**Supplementary Figure S4.** Neighbor-Net graphs based on the distances between melodies of Yaeyama with (a) the parameter set 1 (*δ* = 0.397) and (b) the parameter set 2 (*δ* = 0.3457).


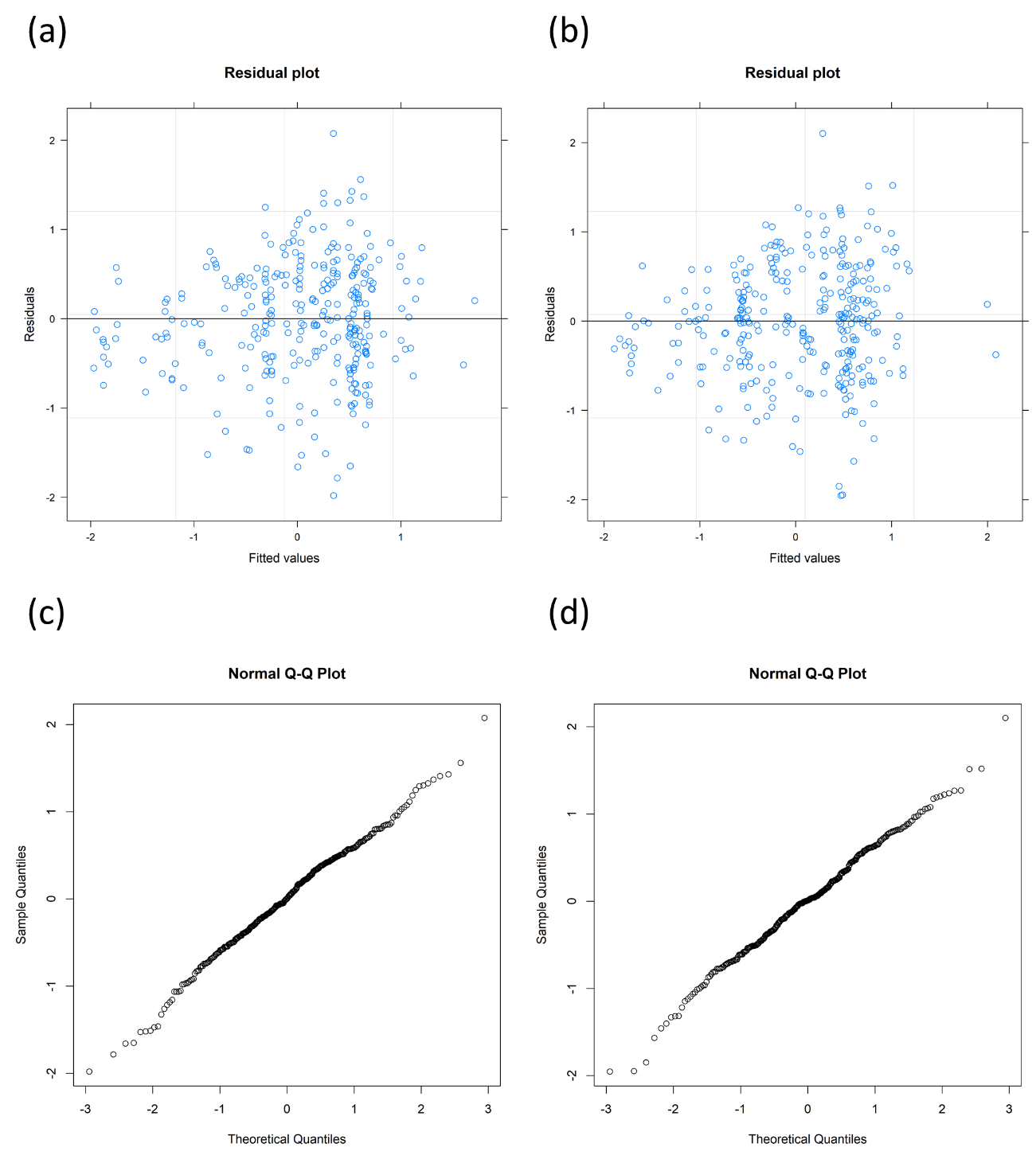


**Supplementary Figure S5.** (a, b) Residual plots for the full model (Eq. (1)) using parameter set (a) 1 or (b) 2. (c, d) QQ plots for the full model (Eq. (1)) using parameter set (c) 1 or (d) 2.
